# Equation-oriented specification of neural models for simulations

**DOI:** 10.3389/fninf.2014.00006

**Published:** 2014-02-04

**Authors:** Marcel Stimberg, Dan F. M. Goodman, Victor Benichoux, Romain Brette

**Affiliations:** ^1^Laboratoire Psychologie de la Perception, CNRS and Université Paris DescartesParis, France; ^2^Département d'Etudes Cognitives, Ecole Normale SupérieureParis, France; ^3^Eaton Peabody Laboratory, Massachusetts Eye and Ear InfirmaryBoston, MA, USA; ^4^Department of Otology and Laryngology, Harvard Medical SchoolBoston, MA, USA

**Keywords:** python, neuroscience, computational neuroscience, simulation, software

## Abstract

Simulating biological neuronal networks is a core method of research in computational neuroscience. A full specification of such a network model includes a description of the dynamics and state changes of neurons and synapses, as well as the synaptic connectivity patterns and the initial values of all parameters. A standard approach in neuronal modeling software is to build network models based on a library of pre-defined components and mechanisms; if a model component does not yet exist, it has to be defined in a special-purpose or general low-level language and potentially be compiled and linked with the simulator. Here we propose an alternative approach that allows flexible definition of models by writing textual descriptions based on mathematical notation. We demonstrate that this approach allows the definition of a wide range of models with minimal syntax. Furthermore, such explicit model descriptions allow the generation of executable code for various target languages and devices, since the description is not tied to an implementation. Finally, this approach also has advantages for readability and reproducibility, because the model description is fully explicit, and because it can be automatically parsed and transformed into formatted descriptions. The presented approach has been implemented in the Brian2 simulator.

## 1. Introduction

Computational simulations have become an indispensable tool in neuroscience. Today, a large number of software packages are available to specify and carry out simulations (Brette et al., 2007). In specifying neural simulations, there is a tension between two often conflicting goals: a simulator should be able to express a wide range of possible models while at the same time allowing the creation of simulations rapidly and easily. Most simulators restrict the basic model components (e.g., neurons and synapses) to a set of predefined standard models, thereby severely limiting the range of possible models.

Defining new model components can be time consuming and technically challenging, requiring the user to implement them in a low-level language such as C++, as in the NEST simulator (Gewaltig and Diesmann, 2007; neuron models can also be specified in NEST's “simulation language interpreter” language), implement them in a special purpose language, as in the NEURON simulator (Carnevale and Hines, 2006) which uses NMODL (Hines and Carnevale, 2000) for this purpose, or to specify them in a complex simulator-independent description language such as NeuroML (Gleeson et al., 2010) or NineML (Raikov et al., 2011). In addition, the definition of model components may not be entirely transparent, as it may be necessary to inspect the simulator source code to know the details of the simulated model. This makes it difficult to assess and reproduce models, and to verify that they correspond to the description of them in a publication.

In this article, we present an approach that combines extensibility with ease-of-use by using mathematical equations to describe every aspect of the neural model, including membrane potential and synaptic dynamics. We implemented this approach in Brian2, the current development version of the Python-based Brian simulator (Goodman and Brette, 2008, 2009), extending previous work which introduced the use of equations-based definitions of membrane potential dynamics. This consistent use of equations to define all aspects of the model greatly extends the scope of Brian, making it possible to run on different computing devices as well as to automatically generate an accurate description of the model to be included in the methods section of a publication.

Simulating a neural model means tracking the change of neural variables such as membrane potential or synaptic weights over time. The rules governing these changes take two principal forms: continuous updates (e.g., the decay of the membrane potential back to a resting state in the absence of inputs) and event-based updates (e.g., the reset after a spike in an integrate-and-fire neuron, or the impact of a pre-synaptic spike on a post-synaptic cell). Generally, continuous updates can be described by deterministic or stochastic differential equations, while event-based updates can be described as a series of mathematical operations. In this unified framework, it is possible to specify a very wide range of model components. With different sets of equations, neuronal models can range from variants of integrate-and-fire neurons to arbitrarily complex descriptions referring to biophysical properties such as ion channels. In the same way, a wide range of synaptic models can fit in this framework: from simple static synapses to plastic synapses implementing short- or long-term plasticity, including spike-timing dependent rules. Finally, mathematical expressions can also be used to describe neuronal threshold conditions or synaptic connections, weights and delays. In sum, this framework based on the mathematical definition of a neural network model seen as a hybrid system allows for expressiveness while at the same time minimizing the “cognitive load” for users, because they do not have to remember the names and properties of simulator-dependent objects and functions but can describe them in a notation similar to the notation used in analytical work and publications (Brette, 2012). In section 2, we describe this general framework and show how neural and synaptic models can be described in this way.

We then explain in section 3 how an equation-oriented description of models can be transformed into runnable code, using code generation. This code generation involves two steps. The first step applies to model components described by differential equations. In most simulators, the numerical integration method (such as forward Euler or Runge-Kutta) is either fixed or part of a model component definition itself. We propose instead to describe the integration method separately, in mathematical notation. Using the capabilities of the Python symbolic manipulation library SymPy (Joyner et al., 2012), we automatically combine this update rule with the actual model equations to yield a sequence of *abstract code* statements. These are high level pseudocode statements that define a sequence of mathematical operations which abstract away the details of how they should be computed (e.g., by loops or vectorised statements), for example **a = b + c**. The second code generation step then applies to this sequence of statements and to all other explicitly given statements and transforms abstract code statements into programming language code (for example in C++) using a general code generation mechanism (Goodman, 2010).

Our approach also has important implications for the issue of reproducibility of simulation results: by making the equations underlying the model fully explicit, the source code also acts as a readable documentation of the model. In addition, giving the neural simulator access to mathematical descriptions of model equations or connection patterns allows for straightforward semi-automatic generation of model descriptions (see e.g., Nordlie et al., 2009), which we describe in section 4.

## 2. Model descriptions

### 2.1. Neural models

Neural models are described by state variables that evolve in time. Mathematically speaking, they are hybrid systems: variables evolve both continuously (e.g., the evolution of the membrane potential between action potentials) and discontinuously through events (e.g., the reset of the membrane potential after a spike in an integrate-and-fire model, or the effect of pre-synaptic spikes). To describe a model therefore requires a system that allows for both of these components. An event is a change in the state variables of the system that is triggered by a logical condition on these variables (possibly after a delay); spikes are the most obvious type of events in neural models. But more generally, there could be other types of events, for example changes triggered when some variable (e.g., intracellular calcium) reaches a threshold value. In addition, it is common that neural models, in particular integrate-and-fire models, have different states, typically excitable and non-excitable (refractory), with different sets of differential equations. An event can then not only trigger changes in state variables but also a transition in state.

It would be possible to make such a system extremely general by allowing for an arbitrary number of general states that a neuron can be in, conditions to change between the states and descriptions of the dynamic evolution within the states (as in NeuroML, Gleeson et al., 2010). Such a system would however have the disadvantage of being very complex to use and to simulate. Therefore, we imposed restrictions so as to simplify the description framework while supporting most neural models currently used.

In the following, we have made the following simplifying choices: (1) there are only two states, active (excitable) and refractory (non-excitable); (2) there is a single type of event per state. In the active state, the only type of event is spikes. It triggers changes in state variables of the neuron (reset) and its target synapses (propagation), and triggers a transition to the refractory state. In the refractory state, the only type of event is a condition that triggers the transition to the active state. This is indisputably restrictive, but was chosen as a reasonable compromise between simplicity and generality. However, the framework could be extended to more general cases (see Discussion).

Finally, another important aspect of neural models is that some state variables represent physical quantities (e.g., the membrane potential, the membrane capacitance, etc.) that have physical units while others correspond to abstract quantities that are unitless. Therefore, to be fully explicit and to avoid any errors when dealing with variables in various units and scales, a description system should allow the user to explicitly state the units in all expressions that deal with state variables.

In sum, the description of a neuron model consists of the following four components: the model equations, the threshold condition, the reset statements and the refractoriness condition. Model equations define all the state variables with their units and describe their continuous evolution in time. The threshold condition describes when an action potential should be emitted and when the reset statements should be executed. Finally, the refractoriness condition describes under which condition a neuron can exit the refractory state. We explain in section 2.1.3 how to specify different dynamics in the refractory state. We will show that this four-component description allows for flexible specification of most neuronal models while being still simple enough to be automatically transformable into executable code.

#### 2.1.1. Model equations

Model equations of point neurons are most naturally defined as a system of ordinary differential equations, describing the evolution of state variables. The differential equations can be non-autonomous (depend on the time *t*) or autonomous, and deterministic or stochastic (referring to one or more stochastic processes). To make the equations more readable, the formalism should also allow for named sub-expressions. Note that even though some models are presented in integral form as sums of post-synaptic potentials, they can be rewritten into a system of equivalent differential equations (see e.g., Destexhe et al., 1994).

As an example, consider the following equations defining a Hodgkin-Huxley model with a passive leak current and active sodium and potassium currents for action potential generation (omitting the equations for the rate variables *h*, *m* and *n*):

$$\begin{array}{l} \frac{\text{d}v}{\text{d}t}=\frac{I_{l}+I_{K}+I_{Na}}{C_{m}}\\ \;I_{l}=g_{l}\left(E_{l}-v\right)\\ I_{Na}=g_{Na}hm^{3}\left(E_{Na}-v\right)\\ \;I_{K}=g_{K}n^{4}\left(E_{K}-v\right) \end{array}$$

Since this model includes the action potential generation in the dynamics it does not need a threshold or reset, except for recording or transmitting spikes.

**Figure 1:**
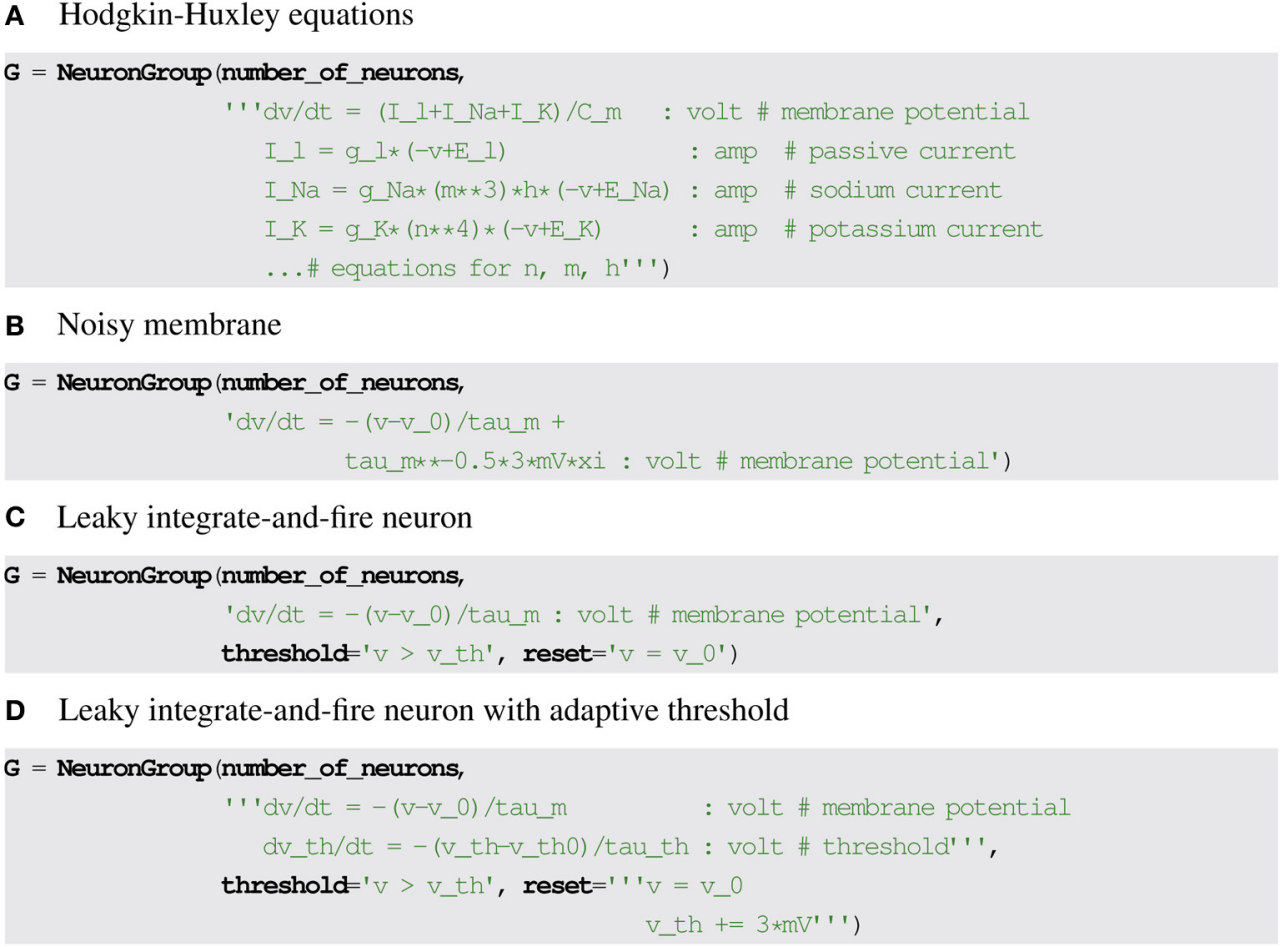
**Examples for neuronal model descriptions in Brian2**. **(A)** Differential equations and parameters are defined via multi-line strings, units are specified after the colon. Note that the units specify the units of the *variables* defined in the respective line which in the case of differential equations is not equivalent to the units of left-hand side and right-hand side of the equations (volt vs. volt/second). **(B)** The symbol xi is used to refer to a stochastic variable for defining stochastic equations. **(C,D)** Threshold conditions and reset statements are also defined by strings.

The model equations can be readily transformed into a string description (including information about the units and general comments), see Figure 1A for a translation into Brian2 syntax, which includes a specification of units after the colon and also uses units (e.g., mV) in the equations themselves.

If a state variable should evolve stochastically, this can be modelled by including a reference to a stochastic white noise process ξ (or several independent such processes ξ_1_, ξ_2_, …). The inclusion of a stochastic process in the model equations means that the differential equations are now stochastic, with important consequences for their numerical integration (see section 3). Figure 1B shows an example for the description of a noisy membrane equation in Brian2.

#### 2.1.2. Threshold and reset

Integrate-and-fire models require a threshold condition and one or more reset statements. A simple leaky integrate-and-fire neuron, for example, can be described as:

$$\begin{array}{l} \;\frac{\text{d}v}{\text{d}t}=-\left(v-v_{0}\right)/\tau_{m}\\ \text{After }v>v_{th}:\;v\leftarrow v_{0} \end{array}$$

where *v*_0_ is the cell's resting and reset potential, *v*_*th*_ is the cell's threshold and τ_*m*_ is the membrane time constant (see Figure 1C for a translation into Brian2 syntax).

Reset statements are not restricted to resetting variables but can perform arbitrary updates of state variables. Similarly, the threshold condition is not restricted to comparing the membrane potential to a fixed value, it is more generally a logical expression evaluated on the state variables. For example, a leaky integrate-and-fire neuron with an adaptive threshold could be described by the equations:

$$\begin{array}{l} \;\frac{\text{d}v}{\text{d}t}=-\left(v-v_{0}\right)/\tau_{m}\\ \;\frac{\text{d}v_{th}}{\text{d}t}=-\left(v_{th}-v_{th0}\right)/\tau_{th}\\ \text{After }v>v_{th}:\;v\leftarrow v_{0}\\ \;v_{th}\leftarrow v_{th}+3\text{mV} \end{array}$$

This model increases the threshold after every spike by 3 mV, between spikes it decays back to the resting value *v*_*th*0_ (see Figure 1D for the Brian2 syntax).

#### 2.1.3. Refractoriness

In integrate-and-fire models, the fact that a neuron is not able to generate a second action potential for a short time after a first one is modeled explicitly (and not following from channel dynamics described in the differential equations). In contrast to that, Hodgkin-Huxley type models only use a threshold for detecting spikes and the refractoriness to prevent detecting multiple spikes for a single threshold crossing (the threshold condition would evaluate to *true* for several time points). In this case, the refractory period is not easily expressed as a time span but more naturally as a condition that the neuron should remain refractory for as long as it stays above the threshold.

**Figure 2:**
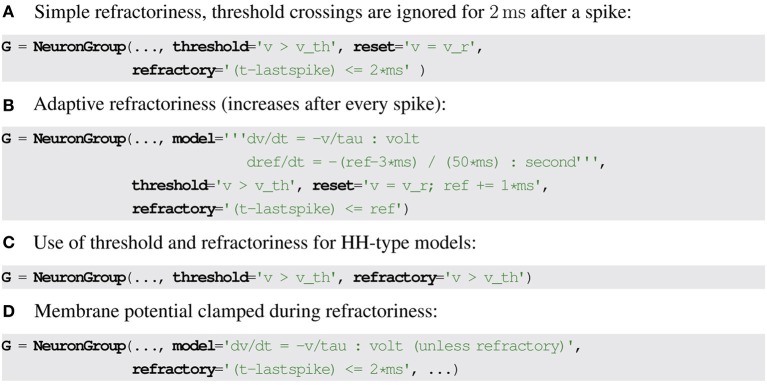
**Examples for refractoriness formulations in Brian2**. **(A,B)** Refractoriness condition based on time since last spike. **(C)** Refractoriness condition based on membrane potential threshold crossing. **(D)** Modifying differential equations based on refractoriness state, the keyword unless refractory clamps a variable *x* during the refractory period, i.e., $\frac{\text{d}x}{\text{d}t}=0$.

A simple formulation of refractoriness that allows some flexibility is to consider that the exit from refractoriness is defined by a logical expression that may depend on state variables and the time of the last spike. In Brian2, the latter is stored in the special variable *lastspike* and the condition evaluates to *false* when the neuron must exit the refractory state. For example, a fixed refractory period of 2 ms can be described as (*t* − *lastspike*) ≤ 2 ms (see Figure 2A for the Brian2 syntax). If the refractoriness should vary across neurons, the expression can refer to a neuronal state variable instead: (*t* − *lastspike*) ≤ *refractoriness*. Since the state variable *refractoriness* can undergo dynamic changes as well (e.g., it could be described by a differential equation or change with every spike as part of the reset), this allows to model very complex refractoriness conditions (Figure 2B). For Hodgkin-Huxley models, the refractoriness condition could simply be *v* ≥ *v*_*th*_, with no reference to the time of the last spike (Figure 2C).

Finally, the set of differential equations could be different in the refractory state. Most generally, this could be described by two different sets of equations. However, neural models generally implement refractoriness in only two ways: either some or all state variables are clamped to a fixed value, or the state variables are allowed to continue to change but threshold crossings are ignored. Only the former case (clamped variables) requires new syntax. We propose to simply mark certain differential equations as non-changing during the refractory period. This can be an explicit syntax feature of the description language (as in Brian2, see Figure 2D). Alternatively, an additional boolean variable representing refractoriness can be introduced, and clamping can then be implemented by multiplying the right hand side of the differential equation by that variable, interpreting the truth values *true* and *false* as 0 and 1, respectively.

### 2.2. Synaptic models

The description of synaptic models has very similar requirements to the description of neuronal models: synaptic state variables may evolve continuously in time and undergo instantaneous changes at the arrival of a pre-synaptic or post-synaptic spike. A synapse connects a pre-synaptic neuron to a post-synaptic neuron, and can have synapse-specific variable values. Events can be pre-synaptic spikes or post-synaptic spikes, and they can trigger changes in synaptic variables, pre-synaptic neural variables or post-synaptic neural variables. With these specifications, describing synaptic models should follow a similar scheme to the one used for neural models: the continuous evolution of the synaptic state variables is described by differential equations, “pre” and “post” statements describe the effect of a pre- or post-synaptic spike. In contrast to neural models, there is no need for a threshold condition since action potentials are emitted from the pre-/post-synaptic neurons according to their threshold conditions.

**Figure 3:**
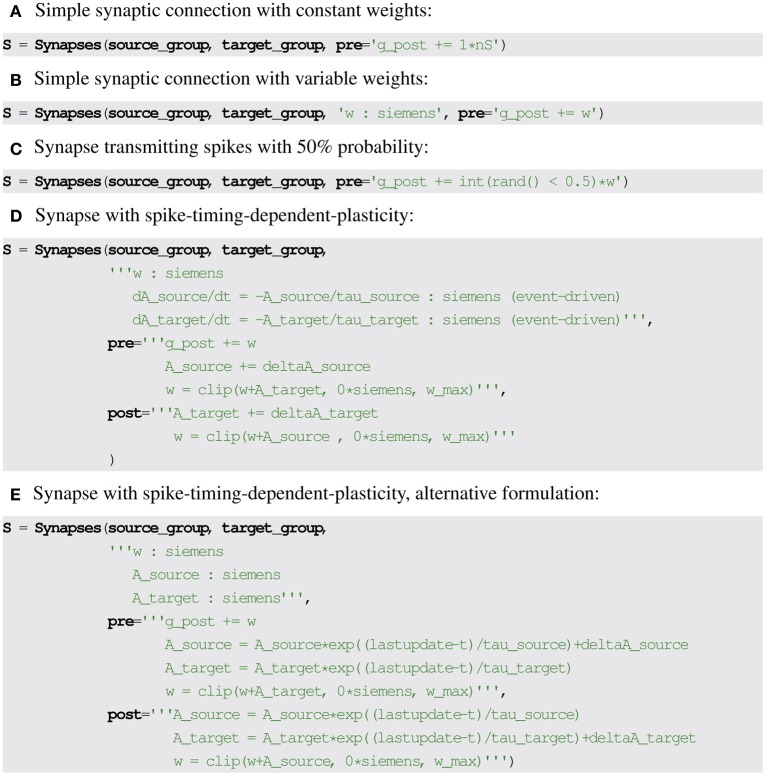
**Examples for synaptic model descriptions in Brian2**. **(A–C)** Forward propagation is described by specifying statements to be executed on the arrival of a pre-synaptic spike (keyword argument pre), changing the value of post-synaptic variables (suffix _post). **(D,E)** Equivalent definitions of spike timing-dependent plasticity using forward and backward propagation. The (event-driven) at the end of the differential equations in **(D)** makes Brian2 automatically convert the equations into the alternative formulation given in **(E)**. The name w_max refers to a constant defined outside of the synaptic model. clip is a function that restricts the values given as the first argument to the range specified by the second and third argument.

A very simple synaptic model might not define any equation and only add a constant value to a post-synaptic conductance or current (or the membrane potential directly) on every pre-synaptic spike, for example *g*_post_ ← *g*_post_ + 1 nS. Note that the index “post” is used to distinguish post-synaptic variables from synaptic variables (in a simple textual description as part of a neural simulator, a suffix _post can be used instead, see Figure 3A). Similarly, the index “pre” can be used for pre-synaptic neural variables. To model differing weights across synapses, a synaptic state variable *w* storing the weights can be introduced, which can then be referred to in the statement: *g*_post_ ← *g*_post_ + *w* (see Figure 3B).

Probabilistic synapses can be modeled by introducing a source of randomness in the “pre” statement. If 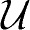
(0, 1) is a random variable uniformly distributed between 0 and 1 and “int” a function converting a boolean expression into a value of 0 or 1, a synapse that transmits spikes with 50% probability can use the following “pre”' statement: *g*_post_ ← *g*_post_ + int(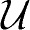
(0, 1) < 0.5) *w* (see Figure 3C).

In the most general formulation, however, the evolution of the synapses' state variables have to be described by differential equations, in the same way as neuronal model equations. By allowing these equations, as well as the “pre” and “post” statements, to refer to pre- and post-synaptic variables a variety of synaptic models can be implemented, including spike-timing-dependent-plasticity and short-term plasticity. For example, models of spike-timing-dependent-plasticity rules make use of abstract “traces” of pre- and post-synaptic activity. Such a model (Song et al., 2000) can be described by the following equations:

$$\begin{array}{l} \;\frac{\text{d}A_{\text{source}}}{\text{d}t}=-A_{\text{source}}/\tau_{\text{source}}\\ \text{After a pre-synaptic }\frac{\text{d}A_{\text{target}}}{\text{d}t}=-A_{\text{target}}/\tau_{\text{target}}\\ \text{ spike: }g_{\text{post}}\leftarrow g_{\text{post}}+w\\ \;A_{\text{source}}\leftarrow A_{\text{source}}+\Delta A_{\text{source}}\\ \text{After a post-synaptic }w\leftarrow \min\left({\left[w+A_{\text{target}}\right]}^{+},w_{\text{max}}\right)\\ \text{ spike: }A_{\text{target}}\leftarrow A_{\text{target}}+\Delta A_{\text{target}}\\ \;w\leftarrow \min\left({\left[w+A_{\text{source}}\right]}^{+},w_{\text{max}}\right) \end{array}$$

where [*x*]^+^ = *x* for *x* > 0 and [*x*]^+^ = 0, otherwise. This assumes an additional state variable *w*, storing the synaptic weights, parameters Δ*A*_source_ and Δ*A*_target_ setting the change in the traces with every spike and a parameter *w*_max_, setting the maximum synaptic weight. The variable *g*_post_ refers to a state variable of the post-synaptic neurons.

Equivalently, the differential equations can be analytically integrated between spikes, allowing for an event-driven and therefore faster simulation (e.g., Brette, 2006; Morrison et al., 2007). It is possible in this case (but not in general) because the variables *A*_source_ and *A*_target_ do not have to be updated at every timestep (which could be very costly if the model involves a large number of synapses) since their values are only needed on the arrival of a spike. Their differential equations are linear, it is therefore possible to include the solutions directly in the “pre” and “post” statements. This requires an additional state variable (*lastupdate* in Brian2) that stores the time of the last state update (pre- or post-synaptic spike). The model can then be reformulated without differential equations (storing *w*, *A*_source_ and *A*_target_ as state variables, see Figure 3E), leading to the formulation of the STDP rule in its integrated form (Song and Abbott, 2001):

$$\begin{array}{l} \text{After a pre-synaptic spike:}\\ \;g_{\text{post}}\leftarrow g_{\text{post}}+w\\ \;A_{\text{source}}\leftarrow A_{\text{source}}\cdot e^{(\text{lastupdate}-t)/\tau_{\text{source}}}+\Delta A_{\text{source}}\\ \;A_{\text{target}}\leftarrow A_{\text{target}}\cdot e^{(\text{lastupdate}-t)/\tau_{\text{target}}}\\ \;w\leftarrow \min\left({\left[w+A_{\text{target}}\right]}^{+},w_{\text{max}}\right)\\ \text{After a post-synaptic spike:}\\ \;A_{\text{source}}\leftarrow A_{\text{source}}\cdot e^{(\text{lastupdate}-t)/\tau_{\text{source}}}\\ \;A_{\text{target}}\leftarrow A_{\text{target}}\cdot e^{(\text{lastupdate}-t)/\tau_{\text{target}}}+\Delta A_{\text{target}}\\ \;w\leftarrow \min\left({\left[w+A_{\text{source}}\right]}^{+},w_{\text{max}}\right) \end{array}$$

The transformation from differential equations to event-driven updates can be done automatically using symbolic manipulation. In Brian2, this is implemented for certain analytically solvable equations, in particular systems of independent linear differential equations (see Figure 3D).

The framework presented so far is insufficient for two important cases, however. One is non-linear synaptic dynamics such as models of NMDA receptors. In this case, individual synaptic conductances must be simulated separately and then summed into the total synaptic conductance. In such a model, the total NMDA conductance of a single neuron can be described as follows (e.g., Wang, 2002):

$$\begin{array}{l} \;g_{\text{total}}^{\text{NMDA}}=\sum_{i}g_{i}^{\text{NMDA}}\\ \;g_{i}^{\text{NMDA}}=w_{i}s_{i}^{\text{NMDA}}\\ \;\frac{\text{d}x_{i}^{\text{NMDA}}}{\text{d}t}=\frac{-x_{i}^{\text{NMDA}}}{\tau_{1}^{NMDA}}\\ \;\frac{\text{d}s_{i}^{\text{NMDA}}}{\text{d}t}=\frac{-s_{i}^{\text{NMDA}}}{\tau_{2}^{NMDA}}+\alpha x_{i}^{\text{NMDA}}(1-s_{i}^{\text{NMDA}})\\ \text{After a pre-synaptic spike at synapse }i:\\ \;x_{i}^{\text{NMDA}}\leftarrow x_{i}^{\text{NMDA}}+1 \end{array}$$

Another important case is gap junctions. In this case, the synaptic current is a function of pre- and post-synaptic potential, which can be expressed in the previously introduced framework, and then all synaptic currents must be added in a neuronal variable representing the total current.

**Figure 4:**
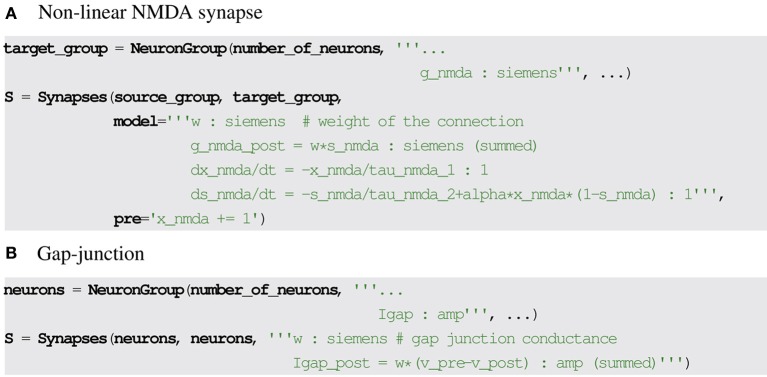
**Examples for synaptic model descriptions involving summed variables in Brian2**. **(A,B)** The equation lines ending with (summed) specify that the post-synaptic variable indicated with the suffix _post is set by summing the corresponding synaptic values on each time step.

Both cases can be addressed by marking every relevant synaptic variable (NMDA conductance, gap junction current) so that the sum over this variable should be taken for all synapses connecting to a post-synaptic neuron and copied over to the corresponding post-synaptic state variable at each simulation time step. See Figure 4 for the corresponding syntax in Brian2.

### 2.3. Network structure

We consider the following specifications for synaptic connections: each synapse is defined by its source (pre-synaptic neuron) and target (post-synaptic neuron); there is a transmission delay from source to synapse, and another one from target to synapse (for the backpropagation needed for example in spike-timing-dependent plasticity rules); there may be several synapses for a given pair of pre- and post-synaptic neurons.

There are several approaches to the problem of building the set of synaptic connections, each having certain advantages and disadvantages. A set of pre-defined connectivity patterns such as “fully connected,” “one-to-one connections,” “randomly connected,” etc. does not allow us to capture the full range of possible connection patterns. In addition, such patterns are not always clearly defined: for example, if neurons in a group are connected to each other randomly, does that include self-connections or not (cf. Crook et al., 2012)? Another approach is to specify connection patterns using a connectivity matrix, allowing for all possible connection patterns. The disadvantage of this approach is that it is not easy to see or report what the underlying connectivity pattern is.

An alternative approach that offers expressivity, explicitness and concise description of connectivity patterns is to use mathematical expressions that specify: (1) whether two neurons *i* and *j* are connected, (2) the probability of connections between neurons *i* and *j*, (3) the number of synapses between *i* and *j* (different types of expressions can be combined).

The first expression is a boolean expression that evaluates to *true* for a certain combination of indices *i* and *j*. For example, *i* = *j* describes a one-to-one connectivity, ⌊*i*/*N*⌋ = *j* describes a convergent pattern where *N* pre-synaptic neurons connect to one post-synaptic neuron and |(*i* − *j* + *N*/2) mod *N* − *N*/2| = 1 describes a ring structure of *N* neurons, where each neuron connects to its immediate neighbours. More complex patterns can be expressed in the same formalism, if the expression can also refer to state variables of the pre- and post-synaptic neurons. Similarly to the synaptic statements described in the previous section, we propose using an index or suffix such as “pre” and “post” to specify these variables.

For example, if the location of neurons in the 2d plane is stored as neural state variables *x* and *y*, spatial connectivity can be readily expressed. The following expression describes a connection from each neuron to all neurons in a 250 μm radius: $\sqrt{{\left(x_{\text{pre}}-x_{\text{post}}\right)}^{2}+{\left(y_{\text{pre}}-y_{\text{post}}\right)}^{2}}<250\text{ μm}$.

The same framework can be used to specify connection probability, possibly in combination with conditions described above. For example, the condition *i* ≠ *j* combined with the probability expression $p_{\text{max}}\;\cdot \;\exp\left(-\frac{{\left(x_{\text{pre}}-x_{\text{post}}\right)}^{2}+{\left(y_{\text{pre}}-y_{\text{post}}\right)}^{2}}{2{\left(125\text{ μm}\right)}^{2}}\right)$ unambiguously defines a structured random connectivity (as a 2-dimensional Gaussian with standard deviation 125 μm) that does not allow for self-connections.

Finally, the expression syntax can also be used to create more than one synapse for a pre-/post-synaptic pair of neurons (useful for example in models where a neuron receives several inputs from the same source but with different delays).

**Figure 5:**
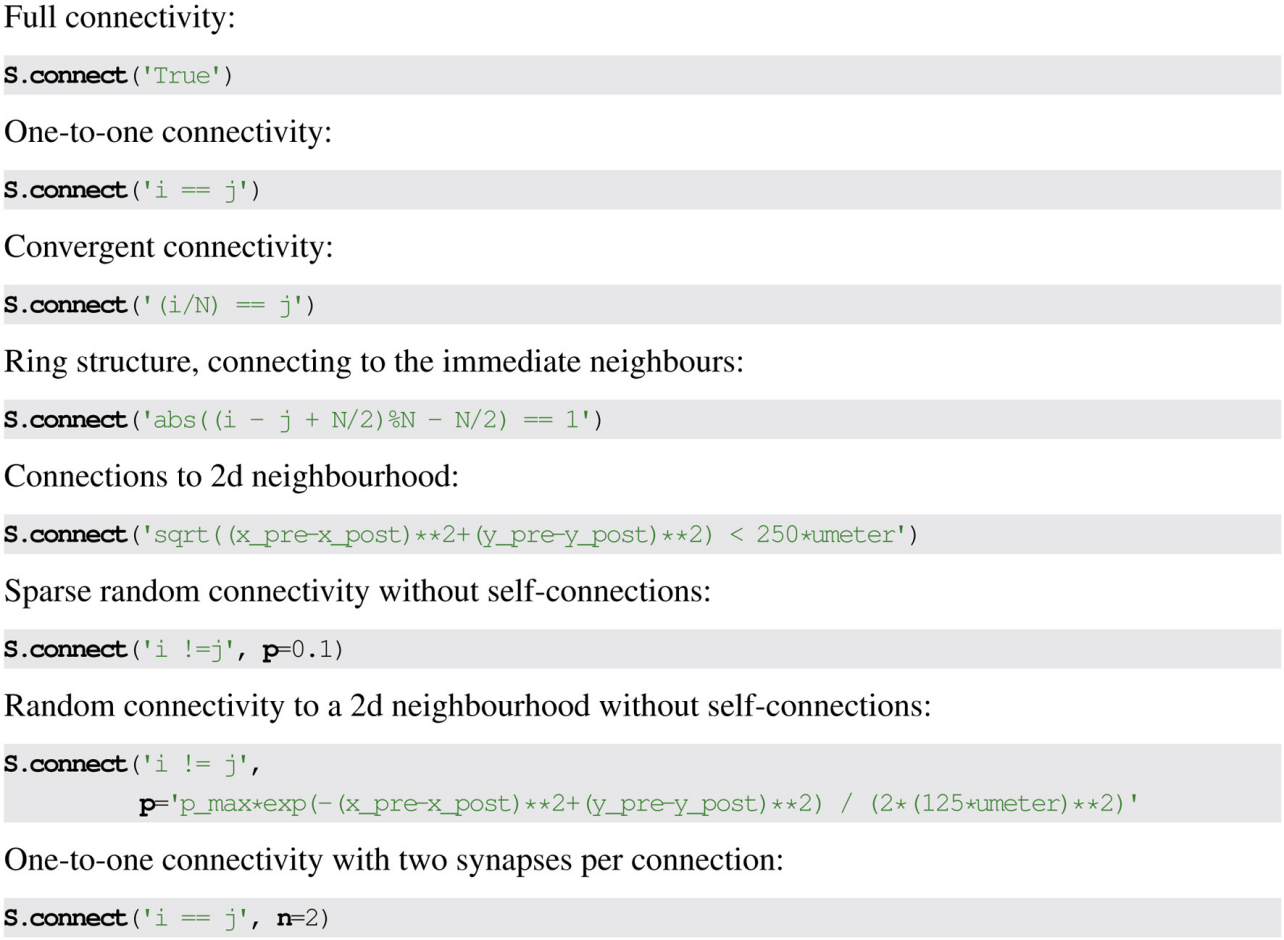
**Examples for synaptic connection descriptions in Brian2**. Strings define conditions based on the pre-synaptic neuron index *i*, post-synaptic neuron index *j*, and pre- and post-synaptic neuron variables with suffix _pre and _post. Optionally, a value or expression for the probability of creating a synapse can be given as the keyword argument p. The number of synapses to create for each connection can be given as the keyword argument n.

See Figure 5 for the use of mathematical expressions to specify synaptic connectivity in Brian2.

### 2.4. Assigning state variable values

**Figure 6:**
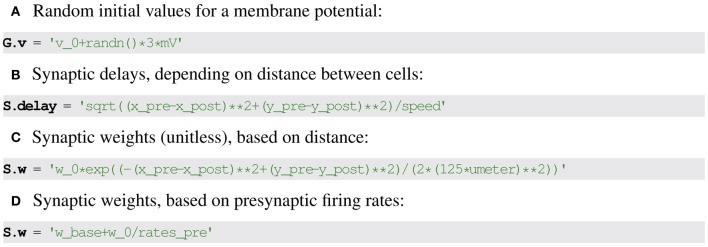
**Examples for specifying initial state variable values in Brian2**. **(A)** Neuronal variables. **(B–D)** Synaptic variables based on pre- and post-synaptic neuronal variables indicated with suffix _pre and _post respectively.

To make the description complete, the initial value of state variables must be set. Many models include state variables that differ across neurons from the start in a systematic (e.g., synaptic weights or delays might depend on the distance between two neurons) or random way (e.g., initial membrane potential values). Such descriptions can be expressed using the very same formalism that has been presented so far (for Brian2 syntax, see Figure 6). For example, initial membrane potential values might be set to random values as *v*_0_ + 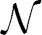
(0, 1) · 3 mV.

For synaptic variables, references to pre- and post-synaptic state variables can be used to express values depending on the neurons that are connected via the synapse. For example, synaptic delays might be set to depend on the distance of the involved neurons as $\frac{\sqrt{{\left(x_{\text{pre}}-x_{\text{post}}\right)}^{2}+{\left(y_{\text{pre}}-y_{\text{post}}\right)}^{2}}}{\text{speed}}$.

Setting state variables with textual descriptions instead of assigning values directly using the programming language syntax may seem to be a questionable choice. However, it offers at least two advantages: firstly, it allows the generation of code setting the variable that then runs on another device, e.g., a GPU, instead of having to copy over the generated values (see section 3); secondly, it allows for a semi-automatic model documentation system to generate meaningful descriptions of the initial values of a state variable (see section 4).

## 3. Generating code from model descriptions

To simulate a neural model means to track the evolution of its variables in time. As shown in the previous section, these dynamical changes consist of three components: *continuous updates* (the model equations), *event-triggered updates* (e.g., the reset in an integrate-and-fire neuron or the synaptic transmission after a pre-synaptic spike), and *logical expressions* defining the events (e.g., a threshold condition).

**Figure 7:**
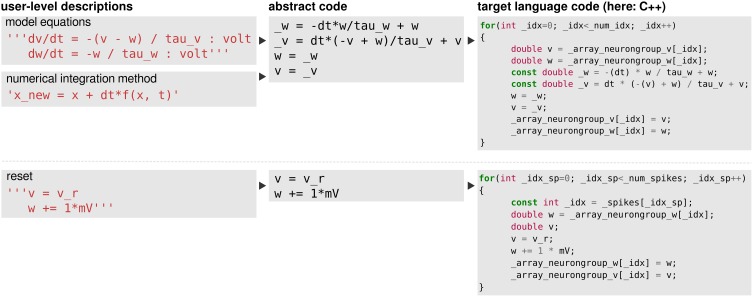
**The code-generation process**. **Left column**: user level descriptions are given either as a set of equations with a numerical integration method, or directly as a sequence of mathematical statements. **Middle column**: in both cases the user level description is turned into a sequence of mathematical statements which we refer to as “abstract code”. This involves no change in the case that the user provided a sequence of statements. **Right column**: the sequence of abstract code statements is converted into valid code in the target language.

Continuous updates are specified in the form of equations that first have to be combined with a numerical integration method to yield abstract code (see Figure 7 top) which is then transformed into programming language code. Event-triggered updates and logical expressions on the other hand are directly specified in the form of abstract code and only have to be transformed into programming language code (Figure 7 bottom). These two steps of code generation will be described in the following.

### 3.1. Numerical integration of continuous state updates

Most neural models are based on equations that are not analytically solvable. The standard approach is therefore to use numerical integration and calculate the values at discrete time points. Many well-studied integration methods exist, allowing for different trade-offs between computational complexity and precision. Often, the provided numerical integration methods are either an integral part of the simulation tool (e.g., in Neuron, Carnevale and Hines, 2006) or built into a specific neural or synaptic model (e.g., in NEST, Gewaltig and Diesmann, 2007).

Here we show a new approach implemented in Brian2, in which a mathematical formulation of an integration method can be combined with the description of the neural model to yield abstract code that is later transformed into target language code using a common “abstract code to language code” framework.

#### 3.1.1. Deterministic equations

Explicit integration methods can be described by a recursive scheme providing the values at discrete time steps. For example, the “midpoint method” (second-order Runge-Kutta) calculates the *n*th value of a variable *x* (where individual values are spaced apart in time by *dt*) according to:

(1) $$x_{n+1}=x_{n}+dt\cdot f\left(x_{n}+\frac{dt\cdot f\left(t_{n},x_{n}\right)}{2},t_{n}+\frac{dt}{2}\right)$$

We specify this integration scheme using the following description, defining a name for a subexpression to avoid nested references to the function *f* (which would make later processing steps considerably more difficult):

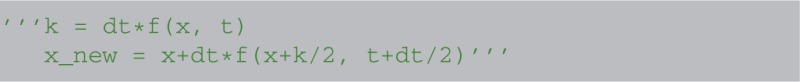



The **x_new** line denotes the final new value for the variable *x*.

Let us consider a model equation with two state variables, describing a neuron with an adaptation current:

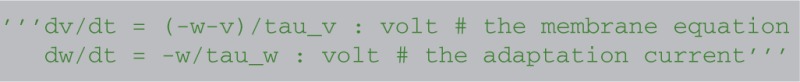



The integration method and the model equations are combined and transformed into abstract code using SymPy, according to algorithm 1.

**Algorithm 1:** Combining model equations and numerical integration method description to yield abstract code. Here, the differential equations in vector form are d*x*/d*t* = *f*(*x*, *t*) where *x* is the vector of state variables with components *x*_*v*_ for variable *v*, and *f* is a vector function with corresponding components *f*_*v*_. All statements in the numerical integration method should be understood as referring to vectors of variables and vector functions.

**for all** statements σ in the numerical integration method **do**
Expand *f*$\left(\underline{\hat{x}},\;\underline{\hat{t}}\right)$ in σ
Replace *x*_new_ in σ by *y*
**for all** state variables *v* **do**
Append component σ_*v*_ of the transformed σ to code
**end for**
**end for**
**for all** state variables *v* **do**
Append *x*_*v*_ = *y*_*v*_ to code
**end for**

Combining the midpoint method and the neuronal equations from above according to this algorithm works as follows:

The model equations in vector form:
  $$\begin{array}{l} \;\underline{x}=\left(v,w\right),\text{with }x_{v}=v,x_{w}=w\\ \underline{f}\left(\underline{x},t\right)=\left(\frac{\left(-x_{v}-x_{w}\right)}{\tau_{v}},\frac{-x_{w}}{\tau_{w}}\right) \end{array}$$
The first statement σ:
  $$\underline{k}=dt\cdot f\left(\underline{x},t\right)$$
Expanding it:
  $$\underline{k}=\left(dt\cdot \frac{-x_{v}-x_{w}}{\tau_{v}},dt\cdot \frac{-x_{w}}{\tau_{w}}\right)$$
*Append to code*:
  $$k_{v}=dt\cdot \frac{-x_{v}-x_{w}}{\tau_{v}}$$
*Append to code*:
  $$k_{w}=dt\cdot \frac{-x_{w}}{\tau_{w}}$$
The second statement σ:
  $${\underline{x}}_{\text{new}}=\underline{x}+dt\cdot f\left(\underline{x}+\underline{k}/2,t+dt/2\right)$$
Expanding it:
  $$\begin{array}{l} {\underline{x}}_{\text{new}}=(x_{v}+dt\cdot \frac{-\left(x_{v}+k_{v}/2\right)-\left(x_{w}+k_{w}/2\right)}{\tau_{v}},\\ \;x_{w}+dt\cdot \frac{-x_{w}+k_{w}/2}{\tau_{w}}) \end{array}$$
After replacing x_new_ by *y*:
*Append to code*:
  $$y_{v}=x_{v}+dt\cdot \frac{-\left(x_{v}+k_{v}/2\right)-\left(x_{w}+k_{w}/2\right)}{\tau_{v}}$$
*Append to code:*
  $$y_{w}=x_{w}+dt\cdot \frac{-x_{w}+k_{w}/2}{\tau_{w}}$$
Finally:
*Append to code:*
  $$x_{v}=y_{v}\text{ and }x_{w}=y_{w}$$

The full abstract code then reads (using names starting with underscores to denote the variables *k* and *y* introduced by the algorithm):

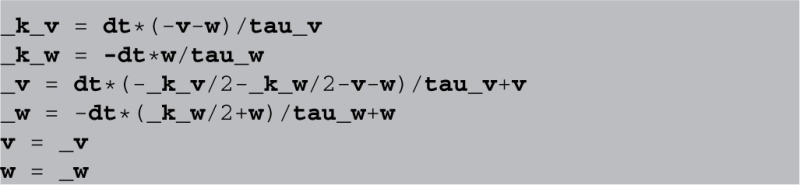



#### 3.1.2. Stochastic equations

The same procedure can also be applied to stochastic differential equations, a description of a state updater in this case looks like (Euler-Maruyama method):

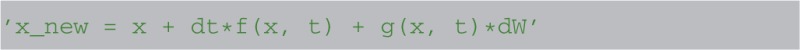



The function *g* in the above formulation corresponds to the factor of the stochastic variable. Note that in the specific case of the Euler-Maruyama method, the function *g* has to be a constant and is therefore not a function of time (“additive noise”), we use the notation *g*(*x, t*) nevertheless for consistency. The symbol **dW** denotes a normally distributed random variable with variance *dt*.

In an equation defining a simple integrate-and-fire neuron with additive noise

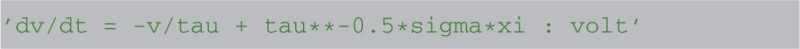



*f* is identified as -**v/tau** and *g* as tau**−0.5*sigma, leading to the following abstract code:

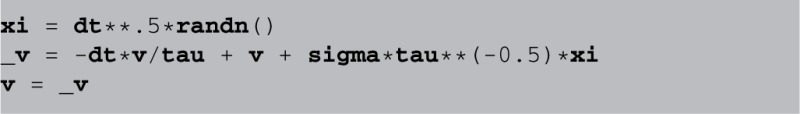



The **randn**() function generates a normal distributed random number.

In the case of more than one stochastic variable (which can be used to model shared noise between variables) the stochastic part of the state updater description is understood as being additive for all stochastic variables. For example, in the case of two stochastic variables, the above described integration method is read as

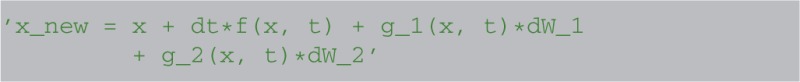



Therefore, the following equation with two stochastic variables

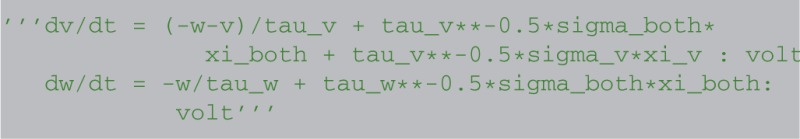



will be integrated as:

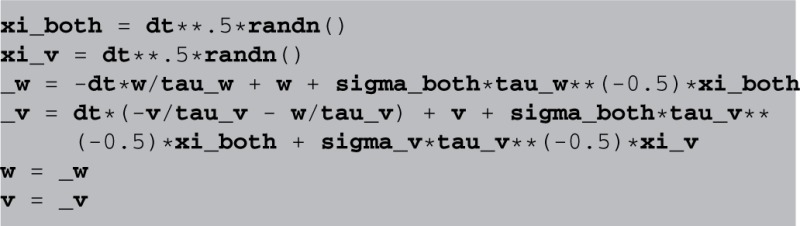



### 3.2. Turning abstract code into runnable code

#### 3.2.1. Abstract code

“Abstract code” is also used for updates that are triggered by specific events, typically a spike, either in a neuron itself or in a pre- or post-synaptic neuron in the context of synapses. In contrast to the model equations, this code is not a mathematical formulation but an explicit statement on how variables should be changed. For example, the reset of an integrate-and-fire neuron might simply state 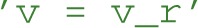 to reset the membrane potential to a specific value. The new value might also depend on the old value, e.g., for an adaptation current: 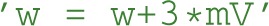. This is a programming language statement and not a mathematical equation, therefore it could also make use of an in-place operator: 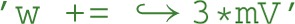. In Brian2, abstract code is used for the reset statements of an integrate-and-fire neuron (where the reset might include updating adaptation variables, changing the refractory period, etc.) and for the code executed on the arrival of pre- or post-synaptic spikes at a synapse. As shown in the previous section, the code generated from model equations via the numerical integration method also is abstract code that will be executed on every timestep.

Abstract code is currently restricted to simple statements of the form:

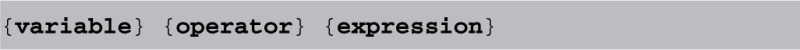



where {**variable**} is the name of a state variable (or a temporary variable used later in the same abstract code block), {**operator**} is one of =, +=, -=, *=, /=, i.e., either an assignment or an inplace operation, and **expression** is an arbitrary expression in Python syntax (which is mostly indistinguishable from C++ syntax, except for rare exceptions such as the absence of a “power” operator in C++, where the **pow** function will be used as a replacement). Even though this code does not allow for control structures such as if statements, some conditional updating can be realized by using boolean expressions. For example, if a model should only update its adaptation variable *w* during an initial time period of 1 s, the reset code could be stated as 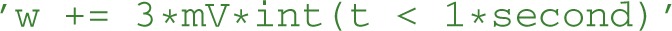 This makes use of an **int** function that converts a boolean expression into 0 or 1.

#### 3.2.2. Expressions

The final remaining building block for model definitions are *expressions*, such as the threshold condition in integrate and fire models. It could refer to a fixed value 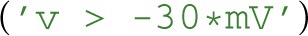, to another state variable 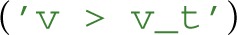 or use any other expression that would be admissible in the right-hand-side of an abstract code statement; in fact, the threshold condition is simply captured in a variable by transforming the expression {**expression**} into the statement _**cond** ={**expression**}. We now describe some specifics for expressions used in making synaptic connections and in assigning state variables.

Building connections from the synaptic connection descriptions presented previously is straightforward: two nested loops iterate over all possible values of *i* and *j*, evaluating the connection condition in turn, using the given *i* and *j* values and accessing any referenced pre-/post-synaptic variables with indices *i* and *j* respectively. If the condition evaluates to *true* and no probability is given, the given number of synapses is created (potentially after evaluating the expression determining the number of synapses, in the same way as the condition was evaluated). If a probability has been given, creating the synapse(s) is only done if a random number drawn from a uniform distribution in the interval [0, 1) is smaller than the given probability (respectively the result of evaluating the given expression).

Note that in languages supporting vectorization (e.g., in Python with the NumPy libraries (Oliphant, 2007)), instead of looping over individual indices, it is more efficient to only loop over one index and use a vector of all values for the other index. In principle it would be possible to have two vectors of all possible index combinations *i* and *j* and not do any looping at all, but this would require generating temporary vectors that have size *N* × *M* (for group sizes *N* and *M*) which is infeasible for large networks.

Setting state variable values with string expressions does not require any specific mechanism and can use the existing code generation techniques. In particular, setting a state variable of a group of neurons can be implemented in the same way as the reset (it can be thought of as a reset that only happens once, not after every spike) and setting state variables of synapses can be implemented in the same way as the effect of a pre- or post-synaptic spike.

#### 3.2.3. Code generation

The abstract code that is generated from the combination of model equations and state updater descriptions or directly provided for event-triggered state updates mostly follows Python syntax conventions but is not directly executable as such. It describes what operations should be executed in the context of a given neuron or synapse, but the implementation may use vectorization or parallelization over neurons/synapses (e.g., in Python, see Brette and Goodman, 2011) or loops (e.g., in C++). Therefore, there is an additional step to transform the abstract code into runnable code for a target language and/or machine.

Let us investigate a simple code statement, resulting from applying forward Euler integration to an integrate-and-fire model with an adaptive current (same example as in the beginning of section 3.1):

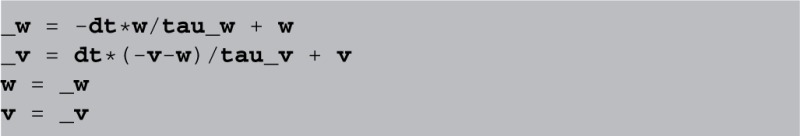



If we let **w** and **v** refer to the state variable arrays (NumPy arrays), these statements are directly executable in Python. However, they don't directly change the original arrays, **w** and **v** instead refer to new temporary arrays that need to be copied back to the original arrays. Python code generation therefore surrounds the above code with:

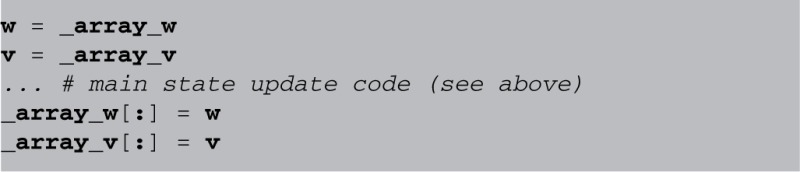



Code in other languages, e.g., C++, does not have built-in support for vectorisation, therefore it has to loop explicitly. Still, the main state update code can be left intact, by surrounding it with^1^:

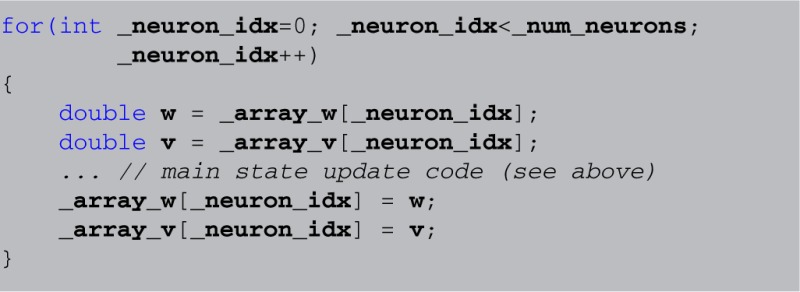



Thus, the transformation from abstract code to target code consists of a model-independent template (responsible for the 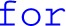 loop in the C++ code), statements for reading/writing state variables from/to the arrays they are stored in and small changes to the abstract code to yield syntactically correct code in the target language (in the case of C++ this includes adding semicolons at the end of statements and replacing x**y by **pow(x, y)**, for example).

More details on the code generation mechanism can be found in Goodman (2010).

## 4. Automatic model documentation

Even though it is now considered best practice for publications in computational neuroscience to make the source code that was used to generate the published results available, a simulator-independent description of the simulation is still valuable. Firstly, it is more accessible, particularly for researchers not familiar with the given simulation environment and/or programming language. Secondly, it simplifies reproducing and verifying the result with other tools.

There are two main approaches to this issue: first, the whole model can be specified in an abstract specification language such as NeuroML (Gleeson et al., 2010) or NineML (Raikov et al., 2011) which then allows the generation of simulator code and textual descriptions, e.g., in the form of an HTML page (see http://opensourcebrain.org for examples). Second, the model may be documented in a standardized form (e.g., Nordlie et al., 2009) that can be directly included in the publication.

The techniques presented in this paper allow for a third approach: since the simulator operates on high-level descriptions of the model in the form of strings, it is possible to create model descriptions automatically. For example, by virtue of SymPy's 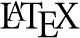 printing facilities, Brian2's **Equations** object can be automatically converted into mathematical descriptions in 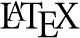 code (shown here in an interactive Python session):

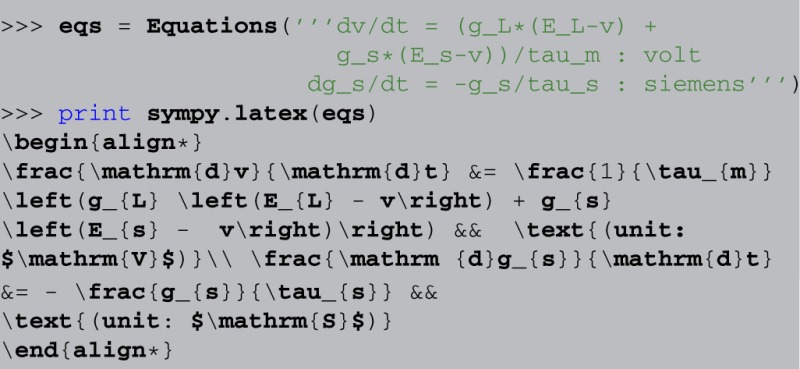



Included in a 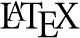 document, this is rendered as:

$$\begin{array}{l} \;\frac{\text{d}v}{\text{d}t}=\frac{1}{\tau_{m}}\left(g_{L}\left(E_{L}-v\right)+g_{s}\left(E_{s}-v\right)\right)\;(\text{unit}:\text{V})\\ \frac{\text{d}g_{s}}{\text{d}t}\text{=}-\frac{g_{s}}{\tau_{s}}\;(\text{unit}:\text{S}) \end{array}$$

This “rich representation” of models not only makes it easier to generate useful model descriptions but can also help in preventing mistakes when generating them; a description that is directly generated from code is always “in sync” with it.

Models are not only defined by their equations, but also by parameter values. For simple parameters, e.g., the time constant τ_*m*_ from above, most simulators would allow for a convenient read-out of the values and therefore be able to display them with name and value in a table, for example. The situation is different for values that have to be described as a vector of values instead of a scalar, e.g., a τ_*m*_ that varies across neurons. Suppose we have a group *G*, consisting of *N* neurons, where we want the membrane time constant τ_*m*_ to vary across neurons. This might be specified by doing:

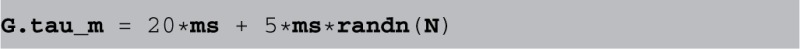



where **randn** generates normally distributed random numbers. All that the simulator “knows” in this case, is that the parameter τ_*m*_ should be set to a given array of *N* numbers. There is no way it can infer where these numbers came from and all it can do in an automatic fashion is either to display all values (which would be inconvenient for large *N*), display a subset of them, to give an “idea” of the numbers used or provide summary statistics, e.g., minimum, maximum, mean and standard deviation of the values. A useful description would have to be manually provided by the researcher, with all the possibilities for making a mistake that this entails.

In contrast, consider the following assignment, providing the expression as a string:

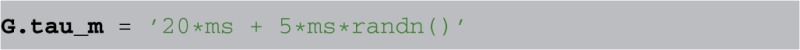



where **randn** is implicitly understood to vectorize over all neurons. Given such a description, the simulator can automatically generate a human-readable documentation of the parameter value, say “τ_*m*_ = 20 ms + 5 ms 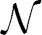
(0, 1),” without any intervention from the researcher.

While a *completely* automatic documentation will not be feasible in all cases, and postprocessing by the researcher is often inevitable, a fully automatic documentation system also offers other advantages: interactive exploration, for example in ipython notebooks (Pérez and Granger, 2007), benefits from having a rich representation of model components. In addition, tools that are concerned with provenance tracking and aim to support the workflow of researchers (e.g., Davison, 2012) could use such a mechanism to give quick and automatic overviews not only over the parameter values used in simulations but also about the model itself, i.e., the equations that define it.

## 5. Discussion

We have described a general framework for defining neural network models, which is based essentially on mathematical equations. It consists of a formalism for defining state variables including their physical units, differential equations describing the dynamics of state variables, conditions on state variables to trigger events and event-triggered statements, changing the state variables discontinuously.

We think that such a mechanism has several advantages over the approach of writing models based on a fixed library of models and mechanisms that can only be extended by writing new descriptions in a low-level language: the equation-oriented framework allows for straightforward descriptions of models; it is explicit about details of the model; by relying on common mathematical notation it does not require the user to learn any special syntax or names used for models and mechanisms.

### 5.1. Limitations

Not all models can be expressed in the framework we have presented, and we will now try to list these limitations. Neuron models were constrained to have only two excitability states, active and refractory, instead of an arbitrary number of states with transitions between them. This is not a fundamental limitation of an equation-oriented approach, but rather a choice that substantially simplifies the syntax.

The framework also neglects the spatial dimension, which would be important to simulate the cable equation on models with an extended spatial morphology, or to simulate calcium dynamics. While a small number of compartments could be already simulated in the current framework (by using the equations for the equivalent electrical model), a complex multi-compartment simulation can only be described in a simple way with an explicit support for this scenario.

Regarding synaptic connections, although a fairly diverse set of synaptic models can be expressed with our framework, there are at least two limitations: structural plasticity and development (requiring the addition and removal of connections during a simulation), and hetero-synaptic plasticity (which cannot be expressed in the presented framework that describes changes in individual synapses independently of other synapses). Extending the framework to cover these cases is not impossible but would require substantial additions.

Finally, the proposed method of specifying the numerical integration method is designed for the general case where the equations cannot be integrated analytically. In Brian2 we do however also allow for special integrators for specific cases such as linear equations that can be integrated exactly using a matrix exponential (Hirsch and Smale, 1974; Rotter and Diesmann, 1999). Even though such integration schemes cannot be expressed in the way described in this paper, they still fit with the general code generation framework: instead of combining the model equations with a textual description of the integration method to yield the abstract code, the numerical integration method is implemented as a Python function that converts model equations directly into abstract code.

### 5.2. Relation to other work

The NineML description language uses string descriptions of differential integrations, conditions and statements in a similar way to the approach presented here. However, due to the use of XML-based definitions and the decision to allow an arbitrary number of states and transition conditions, it is much more verbose in the common use cases and therefore more difficult to use for interactive exploration and rapid development. NineML and the approach presented here are not incompatible but rather complementary. It would be possible to automate the creation of a Brian2 simulation from a NineML description or vice versa.

For describing connectivity patterns, Djurfeldt (2012) proposed the *connection-set algebra*. This is based on similar ideas as the approach presented here, notably it also allows for unambiguous, explicit descriptions and has a high expressiveness. Connection-set algebra builds on the concept of elementary connection patterns (e.g., one-to-one connectivity, full connectivity) that can then be combined to yield more complex patterns. One advantage of this approach over the one we presented is that it allows for an implementation that is very efficient, especially for sparse connectivity patterns such as one-to-one connectivity (it generates all pairs (*i, i*) instead of checking all pairs (*i, j*) for *i* being equal to *j*). However, it also has a few disadvantages compared to our approach: connectivity patterns that are based on arbitrary pre- or post-synaptic state variables are more difficult to specify, and it defines connectivities based on a system that is completely separate from the rest of the model definition and cannot make use of common features such as the unit system.

### 5.3. Future work

The framework presented allows for a wide variety of models with a minimal and unobstrusive syntax. However, we also plan to further increase its expressivity: the restriction to two neural states can be lifted without sacrificing simplicity by supporting multiple event types, each with a condition and a list of statements to be executed. This indirectly allows for an arbitrary number of states, since the state could be represented by a neural variable and equations could then depend on this value. The textual descriptions of numerical integration methods are currently restricted to explicit methods that only refer to the previous simulation time step. The same formalism could be quite naturally extended to implicit methods (e.g., the backward Euler method: *x*_new_ = *x* + *dt* · *f*(*t* + *dt*, *x*_new_) or multistep methods (e.g., the two-step Adams-Bashforth method: $x_{\text{new}}=x+dt\left(\frac{3}{2}f(t,\;x)-\frac{1}{2}f(t-dt,\;x_{\text{prev}})\right)$). More efficient synapse creation for sparse connectivities (e.g., one-to-one connectivity) can be achieved by either analyzing the user-specified connectivity definition for common patterns (e.g., *i* = *j*), or by adding a new syntax explicitly stating which synapses to create (in contrast to a boolean condition for a synapse to exist). Finally, control statements such as 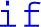 and 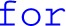 could be added to abstract code. This poses some challenges for code generation, particularly when using vectorised statements, but would allow for even greater expressivity in models, and the use of more complex numerical integration schemes such as variable time step schemes. These additions would allow for a wider variety of expressible models without sacrificing the core principles of model descriptions being explicit, readable and easy to write.

### Conflict of interest statement

The authors declare that the research was conducted in the absence of any commercial or financial relationships that could be construed as a potential conflict of interest.

## Footnotes

^1^The code generated by Brian2 is a bit more complicated, including optimisations using  and _**restrict**_ keywords.
